# Community hydrodynamics created ecological opportunity in Ediacaran shallow marine ecosystems

**DOI:** 10.1093/pnasnexus/pgaf346

**Published:** 2025-10-30

**Authors:** Susana Gutarra, Emily G Mitchell, Rachel L Surprenant, Mary L Droser, Frances S Dunn, Brandt M Gibson, Rachel A Racicot, Simon A F Darroch, Imran A Rahman

**Affiliations:** The Natural History Museum, Cromwell Road, London SW7 5BD, United Kingdom; Department of Zoology, University Museum of Zoology Cambridge, University of Cambridge, Downing Street, Cambridge CB2 3EJ, United Kingdom; Department of Earth and Planetary Sciences, Yale University, 210 Whitney Avenue, New Haven, CT 06511, USA; Department of Earth and Planetary Sciences, University of California, Riverside, 900 University Ave, Riverside, CA 92521, USA; Oxford University Museum of Natural History, University of Oxford, Parks Road, Oxford OX1 3PW, United Kingdom; Department of Agriculture, Geoscience, and Natural Resources, University of Tennessee at Martin, 257 Brehm Hall, Martin, TN 38238, USA; Senckenberg Research Institute and Natural History Museum Frankfurt, Senckenberganlage 25, Frankfurt am Main 60325, Germany; Senckenberg Research Institute and Natural History Museum Frankfurt, Senckenberganlage 25, Frankfurt am Main 60325, Germany; The Natural History Museum, Cromwell Road, London SW7 5BD, United Kingdom; Oxford University Museum of Natural History, University of Oxford, Parks Road, Oxford OX1 3PW, United Kingdom

**Keywords:** Ediacaran, hydrodynamics, computational fluid dynamics, paleoecology

## Abstract

The “second wave” of Ediacaran evolution (∼558–548 Ma) was characterized by the appearance of macroscopic organisms in shallow marine settings, where they formed communities with high morphological and ecological diversity, including new and more complex modes of life. Based on analogy with modern marine ecosystems, these early shallow water communities could have substantially modified local hydrodynamic conditions and influenced resource availability, but we know very little about how they interacted with their fluid environment at larger spatial scales. Here, we use computational fluid dynamics to investigate the hydrodynamics of different shallow marine Ediacaran communities based on fossil surfaces from Russia and South Australia. Our results reveal considerable hydrodynamic variability among these communities, ranging from unobstructed flow, to enhanced mixing, to very low in-canopy flow. This variability represents a noticeable shift from the more conserved hydrodynamic conditions reconstructed for older Ediacaran communities from deep water settings. The variation in how shallow marine Ediacaran communities affected local hydrodynamics could have given rise to notable differences in the distribution of crucial water-borne resources such as organic carbon and oxygen. We therefore hypothesize that increasing variability in community hydrodynamics was an important source of habitat heterogeneity during the late Ediacaran. On long timescales, this heterogeneity may have helped sculpt ecological opportunity, fostering the radiation of animals.

Significance Statement The late Ediacaran was a pivotal time in Earth's history, which saw the radiation of large and complex lifeforms, including some of the first animals. These early animals formed complex seafloor communities tens of millions year before the Cambrian explosion. Here, we use computer simulations of fluid flow to show how Ediacaran shallow water communities influenced their local hydrodynamics in a greater diversity of ways than older deep water communities, contributing to enhanced spatial variability in the distribution of key resources like food and oxygen. We hypothesize that this increasing variation helped create conditions that allowed animals to diversify and evolve new traits, paving the way for the emergence of increasingly complex body plans.

## Introduction

Benthic organisms in marine environments both shape and are shaped by hydrodynamics (1). In modern oceans, communities living on the seafloor exert an influence on the structure of the water column, including current velocity, flow patterns at different spatial scales, and the intensity of vertical and horizontal mixing (2–4). Dense communities of macroscopic organisms in particular can have a powerful effect by baffling currents, concentrating food particles at the sediment–water interface, and creating low-energy refugia (5–7). In turn, the characteristics of fluid flow through and around benthic communities are key to their persistence. Mixing brings oxygen and other dissolved substances vital for respiration and gas exchange, while water currents deliver the nutrients necessary for growth (8–10). Flow structure can also be crucial for reproduction, promoting the dispersal or retention of gametes and larvae (11, 12). The importance of these interactions and feedbacks is increasingly well recognized for large-scale benthic communities such as reefs (13, 14) and marine forests (4), but they remain important at scales down to the individual organism (1, 15).

The complexity of benthic hydrodynamic conditions has changed substantially over the past ∼3.5 billion years, in step with major innovations in the history of life. Following the widespread dominance of stromatolitic reefs for much of the Proterozoic (16), the first macroscopic eukaryotic communities appeared during the late Ediacaran “Avalon” interval (∼574–558 Ma) in relatively deep water settings on continental margins (17). These communities were dominated by frondose forms that interacted with bottom currents to enhance vertical mixing of the surrounding water, thereby promoting gas and nutrient transport (18, 19). During the succeeding “White Sea” interval (∼558–548 Ma), diverse benthic communities appeared in nearshore environments (20, 21). This interval represented the peak in taxonomic and morphological diversity of the so-called Ediacaran macrobiota, and it was characterized by a series of biotic innovations, including motility, burrowing, and new modes of feeding (21–23). This has been referred to as the “second wave” of Ediacaran evolution (21), and it is thought to have paved the way for subsequent radiations (24–27). The colonization of nearshore settings at this time would have posed a range of new physiological and ecological challenges, principally because shallow marine environments typically exhibit greater spatial and temporal heterogeneity (e.g. in terms of seawater chemistry, energy, nutrient levels, sediment dynamics, and temperature) than their deeper marine equivalents (28–30). The hydrodynamics of select White Sea organisms have been investigated using computer simulations of fluid flow, providing some of the oldest evidence for macroscopic suspension feeding in the fossil record (31–34). However, this work has yet to be expanded to the community scale. Consequently, our understanding of organism–fluid interactions and the role they played in driving innovation and escalation during this crucial “second wave” of Ediacaran evolution remains limited.

Here, we investigate the hydrodynamics of Ediacaran White Sea benthic communities for the first time. We digitally reconstruct a variety of fossil assemblages at meso (0.25 and 1 m^2^) scales and perform computer simulations of fluid flow to address several key questions: how did flow conditions vary between different types of communities? How did these conditions differ from older Avalon communities? And to what extent did they reflect the emerging diversity and complexity of animal ecosystems? The results provide valuable insights into how differences in community structure and composition influenced hydrodynamic conditions and thereby shaped ecological opportunity during succeeding phases of evolutionary innovation in the latest Ediacaran and early Cambrian.

## Material and methods

### Material

**Fig. 1.**
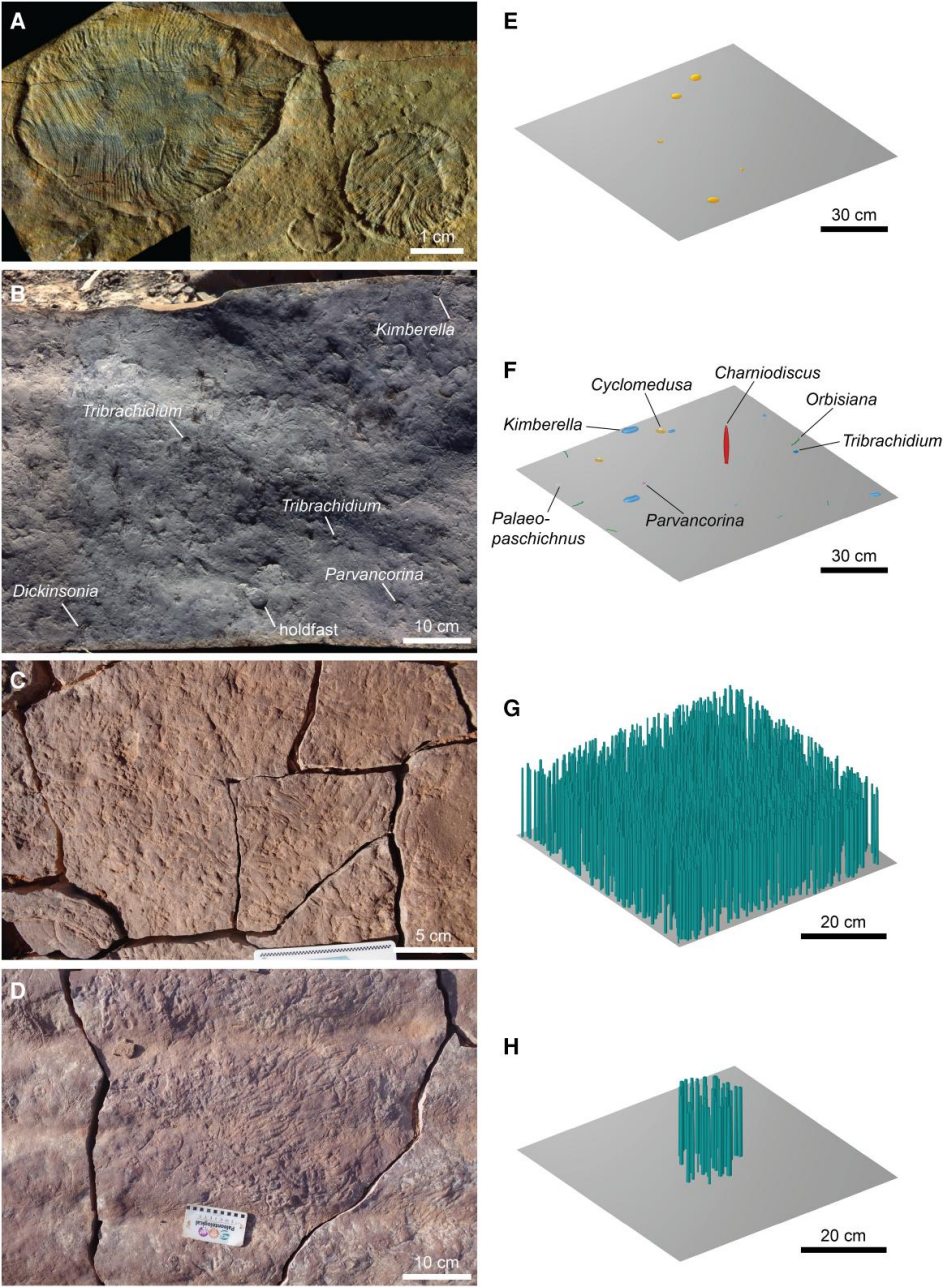
Ediacaran White Sea communities. A) *Dickinsonia* from the DS surface, Konovalovka Member, Cherny Kamen Formation, Sylvitsa River, Central Urals, Russia. Adapted from Mitchell et al. (36). B) *Dickinsonia*, *Kimberella*, *Parvancorina*, and *Tribrachidium* from the KS surface, lower Erga Formation, Winter Coast, White Sea, Russia. Adapted from Mitchell et al. (36). C) Surface-type assemblage of *Funisia* from the WS-MAB surface, Ediacara Member, Rawnsley Quartzite, Nilpena Ediacara National Park, Flinders Ranges, South Australia. D) Cluster-type assemblage of *Funisia* from the TC-BRW surface, Ediacara Member, Rawnsley Quartzite, Nilpena Ediacara National Park, Flinders Ranges, South Australia. E) Simulated DS surface community (sim 1; 1 m^2^ area). F) Simulated KS surface community (sim 1; 1 m^2^ area). G) Simulated FUN5 surface community (sim 2; 0.25 m^2^ area). H) Simulated *Funisia* cluster community (sim 3; 0.25 m^2^ area).

To account for the diversity and disparity among White Sea fossil assemblages (21, 35 ), we investigated a range of surfaces representative of the different community types known from this interval (Fig. 1). We focused on three White Sea-aged fossil surfaces from Mitchell et al. (36): the DS, KS, and FUN5 surfaces. These surfaces were selected because they exhibit differences in the identity of the dominant taxon, species richness, and the number of specimens, and they had been mapped as part of previous work (36), providing the necessary spatial information for modeling virtual communities (see below). They measured 9, 2.74, and 0.78 m^2^ in total mapped area, respectively. The DS surface, dominated by *Dickinsonia* (Fig. 1A), is from the Konovalovka Member of the Cherny Kamen Formation from a site along the Sylvitsa River in the Central Urals, Russia (37). The KS surface preserved a greater diversity of taxa, including *Kimberella*, *Orbisiana*, *Cyclomedusa*, *Charniodiscus*, *Palaeopaschichnus*, *Parvancorina*, and *Tribrachidium* (Fig. 1B); it has been destroyed by weathering and erosion, but came originally from the lower member of the Erga Formation from the Winter Coast of the White Sea, Russia (38, 39). The FUN5 surface is covered in holdfasts of *Funisia* [surface-type assemblage of Surprenant et al. (40)] and was collected from the Ediacara Member of the Rawnsley Quartzite from the Mount Scott Range, Flinders Ranges, South Australia (41, 42). We also examined bedding planes TB-BRW and LV-FUN, which preserve cluster-type assemblages of *Funisia*, from Nilpena Ediacara National Park, South Australia (40). All of these surfaces are thought to comprise assemblages of marine organisms preserved in situ, and they are interpreted as having been deposited in shallow water settings above storm wave base (39, 40, 43, 44).

### Modeling virtual communities

We used the spatial statistics package spatstat (45 ) in R (46) to simulate three virtual communities for each of the DS, KS, and FUN5 surfaces, with the abundance and spatial dynamics of taxa based on published data from Mitchell et al. (36). Previously identified significant associations and interactions (36) were accounted for using heterogeneous Poisson models to model habitat associations within and between taxa (47–49), such as those exhibited by *Funisia* and *Kimberella*, and Thomas Cluster models were used to model dispersal limited reproductive events, as in *Aspidella*. Additionally, we simulated three *Funisia* communities corresponding to the cluster-type assemblages on beds TB-BRW and LV-FUN (40) (Fig. 1D), assuming a mean of 17 individuals (SD of 15) and a diameter of 10–20 cm (SD of 2.5 cm) per cluster (40). The DS and KS surface communities were simulated across areas of 1 m^2^, whereas the FUN5 surface and *Funisia* cluster communities were simulated across areas of 0.25 m^2^. The sizes of these simulated communities represented a balance between the mapped area and taxonomic diversity of the fossil surface and computational limitations due to the size and density of individual organisms [see also Gutarra et al. (19)]. Smaller areas were used for the simulated *Funisia* communities owing to the very high density (>5,000 individuals/m^2^) on the FUN5 surface, which meant it was computationally unfeasible to analyze a larger area.

Simple 3D digital models of *Charniodiscus* (50), *Cyclomedusa* (51), *Dickinsonia* (52), *Kimberella* (53), *Orbisiana* (54), *Palaeopaschichnus* (55), *Parvancorina* (32), and *Tribrachidium* (34) were created using Rhinoceros® v. 7 (56) (Fig. S1). For each taxon, a nonuniform rational basis spline geometry was constructed based on photographs and published reconstructions, informed by personal observations of well-preserved fossil specimens. Fine details such as ridges, frondlets, and fractal structures were omitted to minimize model complexity and thereby economize on computational resources. Previous work has shown that while such features can be important at the scale of individual organisms (see e.g. Olaru et al. (34) and Pérez-Pinedo et al. (57)), they did not strongly influence larger-scale flow patterns (e.g. development of the boundary layer or structure of the wake) and are therefore unlikely to have affected community-scale hydrodynamics (justifying their exclusion in our study). Similarly, static models were used rather than ones able to deform in flow to minimize computational costs.

Virtual communities were assembled in Rhinoceros. For the *Funisia* communities (Fig. 1G and H), the *xy* coordinates generated in R were first imported into the parametric design tool Grasshopper 3D, with individuals modeled as cylinders with a uniform height based on the interpretation of *Funisia* populations as size-similar age cohorts (40, 41). A height of 15 cm was used as this represents an intermediate value recorded for *Funisia* populations (40) (this is likely an underestimate of true height, considering the scarcity of complete individuals). The basal diameter (∼0.2–0.9 cm) was based on the size distribution of holdfasts on the FUN5 surface (36) (FUN5 surface communities) or personal observations of unpublished specimens in cluster-type assemblages (*Funisia* cluster communities); the diameter at the top of each cylinder was 80% of that at the base, accounting for the tapering observed in well-preserved fossil specimens (40, 41). These geometries were then exported into Rhinoceros, with any overlapping cylinders randomly removed.

For the DS and KS surface communities (Fig. 1E and F), models were manually arranged over a surface in Rhinoceros based on the simulated R coordinates, with the dimensions and orientations specified for each model. The dimensions of models in the DS surface communities were based on the size distribution of specimens from the DS surface (36). For the KS surface communities, model dimensions were estimated based on the maximum and minimum sizes of specimens recorded from published photographs of the KS surface (36) and other Russian White Sea fossil-bearing beds (50, 51, 53–55, 58–61); model sizes were obtained from random sampling from a normal distribution within these size ranges, with the exception of *Charniodiscus*, for which holdfast sizes were sampled from a right-skewed distribution [as reported for the holdfast *Aspidella* (59, 62)], with the height then calculated assuming the same ratio between frond and holdfast sizes as seen in the holotype of *Charniodiscus yourgensis* (53). *Charniodiscus* was orientated perpendicular to flow, following the orientation of similar frondose taxa from bedding surfaces in Mistaken Point, Newfoundland, Canada (63) and assuming a mode of life that would have benefited from maximizing the area of the frond exposed to flow (64). Taxa inferred to have been mobile (i.e. *Dickinsonia*, *Kimberella*, and *Parvancorina*) were orientated randomly. *Orbisiana* and *Palaeopaschichnus* were also randomly orientated as the default assumption considering orientation data is not available for these taxa. Lastly, *Cyclomedusa* and *Tribrachidium* are radially symmetrical and thus did not require orientating. The final virtual communities were exported in .STP format.

### Computational fluid dynamics

Computational fluid dynamics (CFD) simulations were performed using COMSOL Multiphysics v. 5.6 (65) following established protocols (19, 64, 66). Virtual communities were imported into COMSOL and placed at the bottom of the computational domain, which consisted of a cuboid measuring 3 m in length, 1.5 m in width, and 0.45 m in height (Fig. S2A). This was sufficiently large to allow flow to fully develop around all the simulated communities. The models were subtracted from the domain using a Boolean operation, with the standard material properties of water (density *ρ* = 1,000 kg/m^3^ and dynamic viscosity *μ* = 0.001 kg/s·m) assigned to the space surrounding the models. An inlet with fully developed flow was specified at one end of the domain and an outlet with a static pressure of 0 Pa was defined at the opposing end. No-slip boundaries were assigned to the models and the lower surface of the domain, with slip boundaries used for the upper surface of the domain and periodic flow conditions (with a pressure difference of 0 Pa) for the sides of the domain.

The inlet velocity was inferred based on the sediment grain size and bedforms described for the DS, KS, FUN5, TB-BRW, and LV-FUN surfaces (36, 39 , 40, 42), which allowed us to estimate bottom current velocities of between 0.1 and 0.4 m/s using the bedform-velocity matrix of Stow et al. (67). These flow velocities are also consistent with typical values recorded in analogous modern shallow marine settings (68–70). CFD simulations were performed for all virtual communities using an average inlet velocity of 0.2 m/s. We also carried out simulations of average velocities of 0.1 and 0.4 m/s for select communities to assess the impact of flow velocity on the results. 3D, incompressible flow was simulated using a stationary solver to compute the Reynolds averaged Navier–Stokes equations using the Spalart–Allmaras turbulence model (71). Additionally, select simulations were repeated using the K-epsilon (k-ε) turbulence model (72) to allow us to visualize the turbulent kinetic energy.

The domain was meshed using six layers of prismatic elements along the no-slip boundaries and tetrahedral elements in the rest of the domain. A refinement area was created around the virtual community, which measured at least 1.5× the height of the tallest model (a minimum height of 0.2 m was used). Mesh size settings (maximum element size = 1.8 mm, minimum element size = 0.03–0.196 mm, maximum element growth rate = 1.1, curvature factor = 0.4, resolution of narrow regions = 0.9 in the refinement area; maximum element size = 4.9 mm, minimum element size = 1.4–1.47 mm, maximum element growth rate = 1.2, curvature factor = 0.7, resolution of narrow regions = 0.6 in the rest of the domain) largely followed Gutarra et al. (19), with the minimum element size modified based on the number and complexity of the modeled organisms.

### Sensitivity tests

Both the size and density of individuals can vary considerably among surface-type assemblages of *Funisia* (40). To explore the sensitivity of our results to the modeled height of *Funisia*, we carried out three CFD simulations for one of the FUN5 surface communities (sim 2) with model heights changed to (i) 12 cm, (ii) 18 cm, and (iii) varying between 12 and 18 cm. Additionally, to assess the impacts of population density on the results, we ran CFD simulations of the three FUN5 surface communities with model density reduced from ∼5,000 individuals/m^2^ to ∼900 individuals/m^2^ [consistent with the lowest densities reported for surface-type assemblages (40)] by randomly removing models from the original virtual communities. These sensitivity tests were performed with the same settings as the main analyses, using an average inlet velocity of 0.2 m/s.

### Visualization and quantification

CFD results were visualized in COMSOL as 2D plots (horizontal cross-sections) of streamwise velocity (*u*) and vertical velocity (*w*) normalized by the average inlet velocity (*U*_0_) and 3D flow streamlines. We also visualized 2D plots and 3D isosurfaces of turbulent kinetic energy (*k*) normalized by *U*_0_^2^ for select communities.

Streamwise velocities (*u*) were sampled from 20 evenly spaced lines at the back of the community (Fig. S2B) and from a central line at the inlet (Fig. S2C). Additionally, vertical (*w*) velocities were sampled from a 3D grid of evenly spaced points. In the DS and KS surface and *Funisia* cluster communities, the point grid surrounded the entire virtual community (Fig. S2D). In the FUN5 surface communities, to eliminate the strong influence from the leading edge [where flow conditions are different from the bulk of the community (73)], *w* values were sampled from the fully developed region towards the back of the domain (‘Subsample 2' in Fig. S3; see Supplementary text for further details). Plots were made in R using the package ggplot2 (74).

## Results

**Fig. 2.**
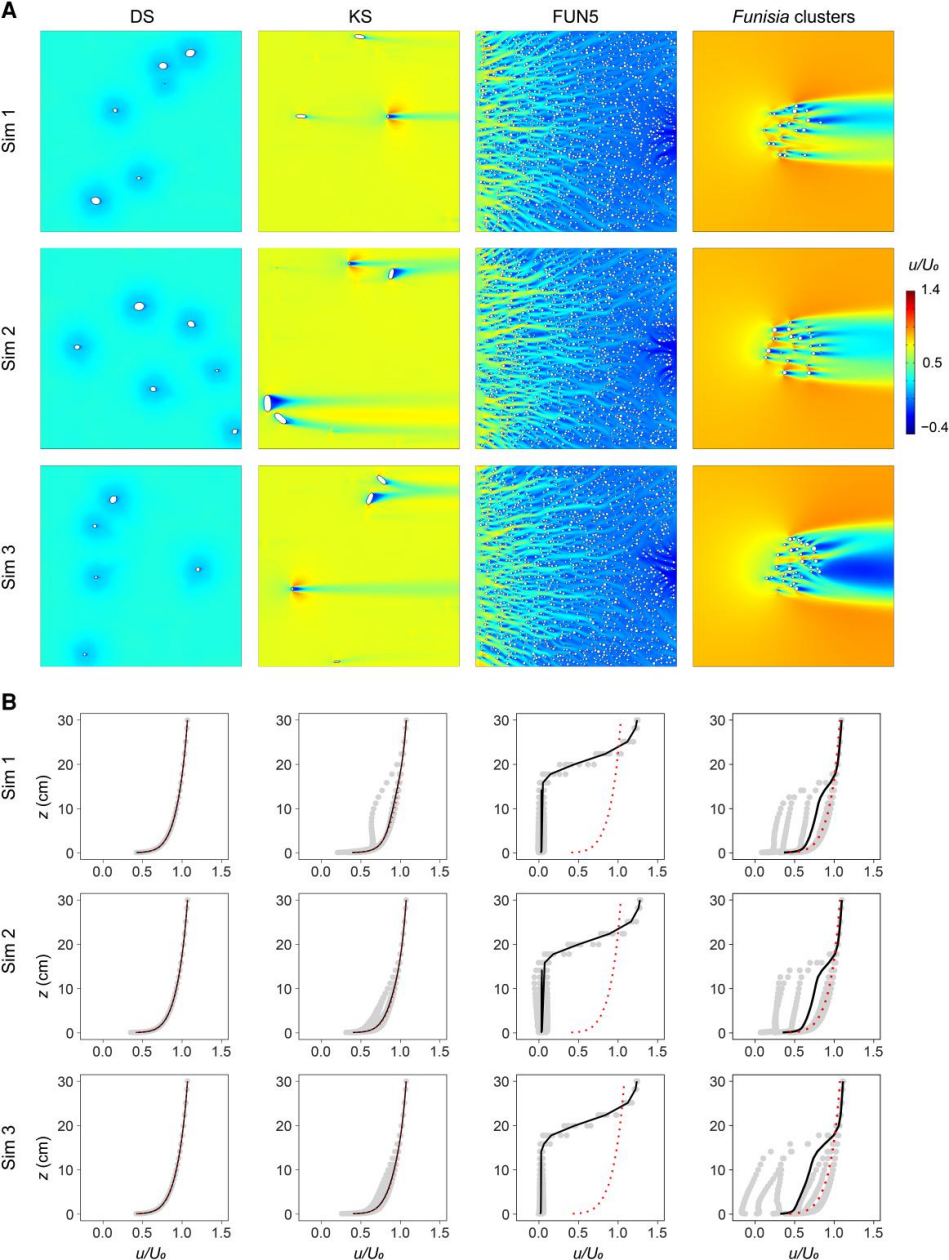
Plots of streamwise velocity for White Sea communities. CFD results for three simulated communities (sim 1–sim 3) of the DS (1 m^2^ area), KS (1 m^2^ area), FUN5 (0.25 m^2^ area) surfaces, and *Funisia* clusters (0.25 m^2^ area). A) 2D plots of streamwise velocity (*u*) relative to the average inlet velocity (*U*_0_ = 0.2 m/s) for horizontal cross-sections at heights *z* = 0.05 cm (DS), *z* = 1 cm (KS), and *z* = 5 cm (FUN5 and *Funisia* clusters). Direction of ambient flow from left to right. B) Plots of streamwise velocity (*u*) relative to the average inlet velocity (*U*_0_ = 0.2 m/s) at heights between *z* = 0 and *z* = 30 cm. The black line shows the mean streamwise velocity and the dotted red line shows the undisturbed boundary layer profile.

**Fig. 3.**
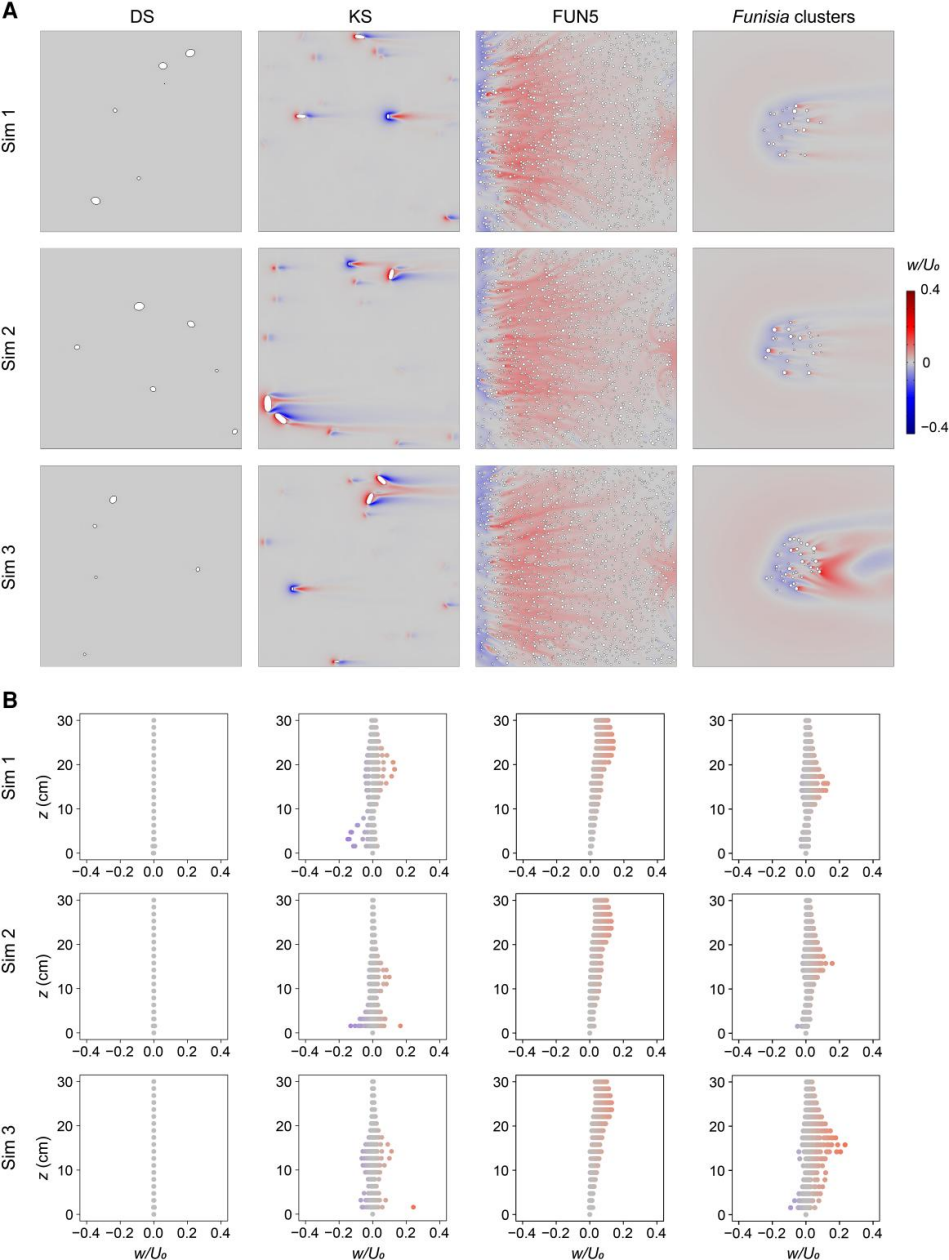
Plots of vertical velocity for White Sea communities. CFD results for three simulated communities (sim 1–sim 3) of the DS (1 m^2^ area), KS (1 m^2^ area), and FUN5 (0.25 m^2^ area) surfaces and *Funisia* clusters (0.25 m^2^ area). A) 2D plots of vertical velocity (*w*) normalized by the average inlet velocity (*U*_0_ = 0.2 m/s) for horizontal cross-sections at heights *z* = 0.05 cm (DS), *z* = 1 cm (KS), and *z* = 5 cm (FUN5 and *Funisia* clusters). Direction of ambient flow from left to right. B) Plots of vertical velocity (*w*) normalized by the average inlet velocity (*U*_0_ = 0.2 m/s) at heights between *z* = 0 and *z* = 30 cm.

**Fig. 4.**
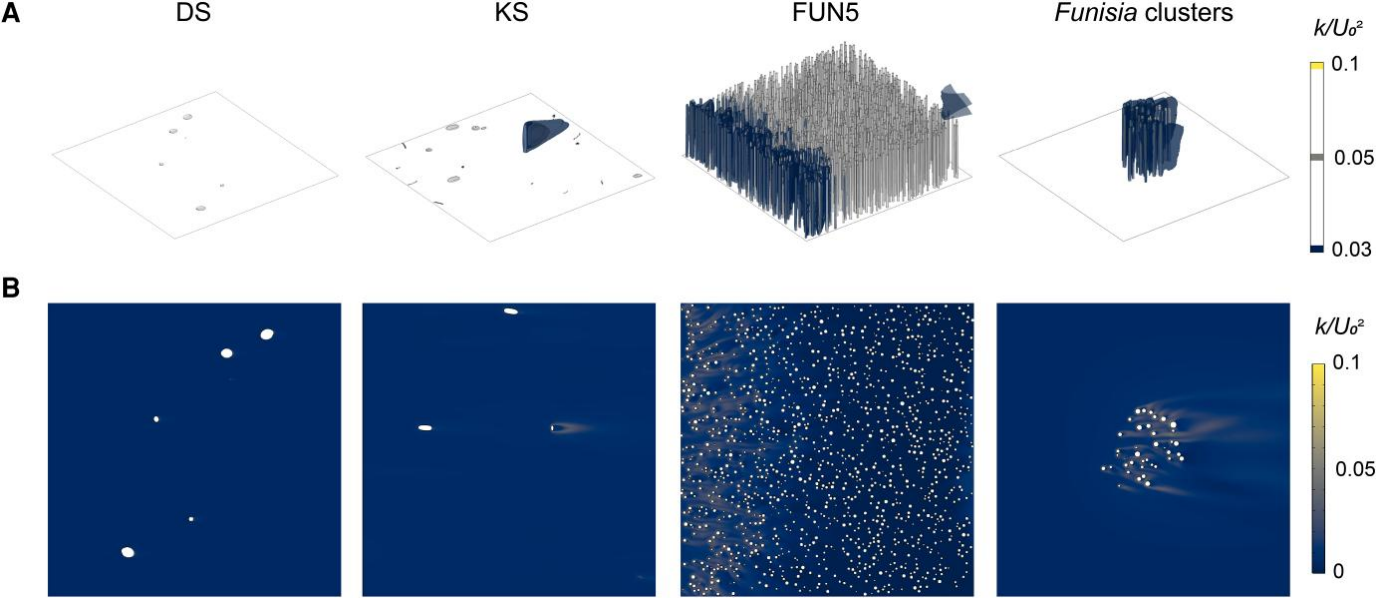
Plots of turbulent kinetic energy for White Sea communities. CFD results for one simulated community of the DS (sim 1; 1 m^2^ area), KS (sim 1; 1 m^2^ area), FUN5 (sim 2; 0.25 m^2^ area) surfaces, and *Funisia* clusters (sim 3; 0.25 m^2^ area). A) 3D isosurfaces of turbulent kinetic energy (*k*) normalized by the average inlet velocity (*U*_0_ = 0.2 m/s) squared. B) 2D plots of turbulent kinetic energy (*k*) normalized by the average inlet velocity (*U*_0_ = 0.2 m/s) squared for horizontal cross-sections at heights *z* = 0.05 cm (DS), *z* = 1 cm (KS), and *z* = 5 cm (FUN5 and *Funisia* clusters). Direction of ambient flow from left to right.

Our CFD simulations revealed considerable variation in the hydrodynamics of different White Sea community types, consistent across all simulated inlet velocities (Figs. 2–4 and S4–S6). In the DS surface communities, flow was not strongly influenced by the modeled organisms, which produced only very short wakes immediately downstream of models (Figs. 2A, S4, S5A, and S6A). The modeled organisms in the KS surface communities had a greater impact on flow patterns, diverting the flow laterally and vertically and creating long wakes (Figs. 2A, S4, S5A, and S6A). In the FUN5 surface communities, flow decelerated from where it first encountered the community, becoming fully developed at a distance of ∼30 cm from the leading edge, at which point there was very little flow between the modeled organisms (Figs. 2A, S4, S5A, and S6A). In this fully developed region, there was strong interaction between the wakes produced by neighboring models, meaning that individual wakes could not be recognized (Figs. 2A, S4, S5A, and S6A). Lastly, in the *Funisia* cluster communities, flow was diverted around the sides and over the top of the community, as well as moving through it; the extent of throughflow varied depending on the density of the modeled organisms, which created wakes that interacted with each other (Figs. 2A, S4, S5A, and S6A).

In the DS and KS simulated communities, the mean normalized streamwise velocity (*u/U*_0_) increased logarithmically with height, giving a velocity profile that was almost indistinguishable from the undisturbed boundary layer (Figs. 2B, S5B, and S6B). This was markedly different to the FUN5 surface and *Funisia* cluster communities, where the streamwise velocity was slowed relative to the undisturbed boundary layer profile until it reached the top of the community, with a strong velocity gradient developed above this (Figs. 2B, S5B, and S6B).

In the DS surface communities, the normalized vertical velocities (*w/U*_0_) were close to zero, whereas the KS surface and *Funisia* cluster communities displayed positive and negative vertical velocity perturbations in the vicinity of the modeled organisms (Figs. 3A, S5C, and S6C), giving roughly bottle-shaped vertical velocity plots (Figs. 3B, S5D, and S6D). Within the fully developed region in the FUN5 surface communities, vertical velocities were generally small (Figs. 3A, S5C, and S6C), with a notable positive perturbation occurring above the community (Figs. 3B, S5D, and S6D).

The normalized turbulent kinetic energy (*k*/*U*_0_^2^) was very low in the DS surface communities and in the fully developed region in the FUN5 surface communities (Fig. 4). In contrast, turbulent kinetic energy was enhanced in the KS surface communities, with turbulence produced in the wakes of models with larger frontal areas (Fig. 4). Similarly, there was elevated turbulent kinetic energy in the *Funisia* cluster communities, both surrounding the models and within their wakes (Fig. 4).

Our sensitivity tests demonstrated that the flow patterns obtained for the FUN5 surface communities were largely unaffected by model height (Fig. S7), with a steep gradient in mean normalized streamwise velocity (Fig. S7A) and positive perturbations in normalized vertical velocity (Fig. S7B) always occurring just above the maximum height of the community. Model density had a stronger influence on the results, with the low-density communities allowing greater throughflow and showing a weaker leading-edge effect compared to the original simulated communities (Fig. S8). Moreover, the heights at which the streamwise velocity gradient (Fig. S8B) and vertical velocity perturbations (Fig. S8D) occurred were lower than in the original simulated communities (Fig. S8A and C).

## Discussion

Our analyses demonstrate that different types of White Sea communities had distinct flow conditions. The DS surface communities did not substantially modify the ambient hydrodynamics, with flow velocities and turbulence in the bottom boundary layer largely unaffected by the presence of the modeled organisms. The KS surface communities had a greater influence on flow, generating enhanced vertical mixing (i.e. strong perturbations in vertical velocity) and turbulence, but still produced mean streamwise velocity profiles that closely resembled an undisturbed boundary layer (75). Lastly, the FUN5 surface and *Funisia* cluster communities were characterized by the development of a roughness sublayer, where streamwise velocities were reduced, with an inflection point (maximum velocity gradient) at the top of the community, both features of canopy flow (4, 76). However, in the FUN5 surface communities, positive vertical velocity perturbations were only evident above the community, whereas the *Funisia* cluster communities created stronger patterns of vertical mixing and turbulence that were more similar to the KS surface communities. Thus, shallow marine environments inhabited by benthic communities during the White Sea interval would have been associated with a range of hydrodynamic conditions.

**Fig. 5.**
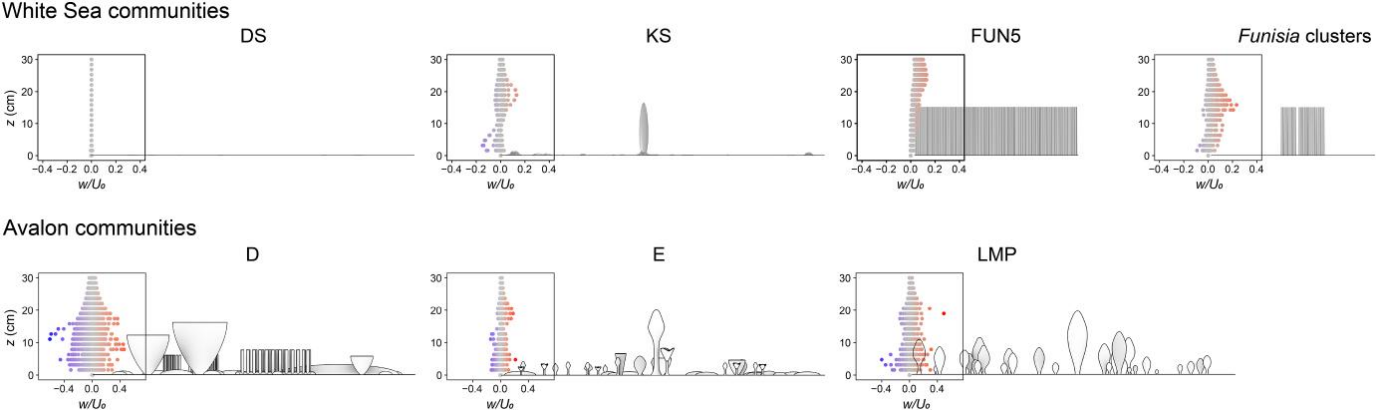
Plots of vertical velocity for White Sea and Avalon communities. CFD results for one simulated community for the DS (sim 1; 1 m^2^ area), KS (sim 1; 1 m^2^ area), and FUN5 (sim 2; 0.25 m^2^ area) surfaces, *Funisia* clusters (sim 3; 0.25 m^2^ area), and the D (sim 2; 1 m^2^ area), E (sim 2; 1 m^2^ area), and LMP (sim 2; 1 m^2^ area) surfaces. Plots of vertical velocity (*w*) normalized by the average inlet velocity (*U*_0_ = 0.2 m/s) at heights between *z* = 0 and *z* = 30 cm, with models of the corresponding community shown in frontal view.

Notably, we find that White Sea communities were characterized by greater variation in hydrodynamics than those from the earlier Ediacaran (Avalon) (Fig. 5). Previous analyses of ∼565-million-year-old Avalon communities found they enhanced mixing of the surrounding seawater (19), similar to some present-day marine animal forests (4). We see this feature in two of the studied White Sea community types. However, other flow conditions reconstructed for our White Sea communities (e.g. flow at very high canopy densities) are unknown from Avalon communities and could thus represent their first appearance, indicative of a step increase in hydrodynamic variability coincident with the emergence of new body plans and behaviors (21, 22).

These results allow us to develop hypotheses for how community structure and hydrodynamics shaped resource availability in late Ediacaran shallow water settings. The DS surface communities were composed of widely spaced, low-relief organisms (i.e. *Dickinsonia*) that had minimal impact on flow and would not be expected to have greatly affected the transport of dissolved and particulate substances (4). This is consistent with the inference that *Dickinsonia* fed via the external digestion of benthic microbial mats (77, 78) and was therefore not reliant on water-borne nutrients as a main food source. In contrast, the KS surface communities comprised a much wider diversity of organism shapes and sizes, creating locally enhanced vertical mixing and turbulence that would have served to redistribute resources like oxygen and organic carbon, as can be seen in modern communities of benthic macroinvertebrates (4, 8–10). The KS surface communities included probable low-level suspension feeders [e.g. *Tribrachidium* (31, 34)], which may have been able to take advantage of the increased vertical transport of suspended particulate organic matter brought about by turbulent mixing. *Funisia* occurred in two types of communities characterized by distinct flow conditions. Discrete clusters of *Funisia* promoted vertical mixing of the surrounding water, thereby enhancing gas and nutrient transport in a similar manner to the KS surface communities. However, dense communities of *Funisia* covering large areas of the seafloor were associated with very low in-canopy flow, likely resulting in greatly reduced mass transfer rates and the deposition of suspended particles, as seen in modern marine canopies (4, 6, 73). This variation in how different *Funisia* community types affected the distribution of resources suggests they did not require specific flow conditions for feeding or respiration.

Late Ediacaran shallow water communities modified hydrodynamics in a greater range of ways than older deeper water communities, likely reflecting changing environmental pressures [e.g. moving away from the deep marine stenothermal cradle of Boag et al. (30)] and/or the evolution of new tissues and modes of life that differed in their reliance on water-borne resources (22). In this regard, the increased disparity in flow conditions likely mirrored the increasing ecological complexity of early animals. We hypothesize that the generation of hydrodynamic variability across White Sea benthic communities had the potential to be a powerful ecosystem engineering process influencing the distribution of nutrients and other resources, similar to the present day (4), and this may plausibly have shaped ecological opportunity on longer timescales. For example, the mixing produced by the KS surface and *Funisia* cluster communities could have increased the supply of suspended food particles to communities, potentially leading to an expansion in suspension feeding strategies (33), promoting horizontal habitat heterogeneities (36), and cementing resource hotspots on the seafloor (27, 79). In contrast, reduced rates of mass transfer in the FUN5 surface communities would have limited the supply of suspended resources, while promoting the settling of organic and inorganic particles.

Previous work on White Sea-aged surfaces has emphasized the extent and spatial heterogeneity of seafloor microbial mats, and the crucial role these would have played as a food source for early animals (36, 80, 81). Our results contribute to this picture, suggesting that increasing hydrodynamic variability among local communities may have created regional heterogeneity in both the character and availability of nutrients. Together, these sources of habitat heterogeneity may have helped sculpt ecological opportunity during the “second wave” of Ediacaran evolution (27, 79). With the growing recognition that ecosystem engineering effects can sometimes scale upwards to impact macroevolutionary patterns (82–85), this raises the possibility that these processes played an important role in the radiation of macro-organisms with increasingly complex lifestyles, including early bilaterians.

## Supplementary Material

pgaf346_Supplementary_Data

## Data Availability

Spreadsheets with details of the simulated communities, digital models, and CFD results files are available at Zenodo: https://doi.org/10.5281/zenodo.17035671. The code used for spatial ecological modeling is available at GitHub: https://github.com/egmitchell/WhiteSeaSpatialSimulations.
