# Supplementary material for: Community hydrodynamics created ecological opportunity in Ediacaran shallow marine ecosystems
Source: PNAS Nexus. 2025 Oct 30;4(11):pgaf346. doi: 10.1093/pnasnexus/pgaf346 (PMC12619063; doi:10.1093/pnasnexus/pgaf346)
Supplement: pgaf346_Supplementary_Data [file pgaf346_supplementary_data.pdf]

PNAS nexus

Supplementary Information for
Community hydrodynamics created ecological opportunity in Ediacaran shallow marine ecosystems

Susana Gutarra, Emily G. Mitchell, Rachel L. Surprenant, Mary L. Droser, Frances S. Dunn, Brandt M. Gibson, Rachel A. Racicot, Simon A. F. Darroch and Imran A. Rahman

Imran A. Rahman
Email: imran.rahman@nhm.ac.uk

This PDF file includes:

Supplementary text
Figures S1 to S8
SI References

Supplementary Information Text

Supplementary methods

To determine the region in which the flow became fully developed in the FUN5 surface communities, streamwise and vertical velocities were sampled for the entire domain and different subsamples towards the back of the domain for one simulated community (Sim 1) using an average inlet velocity of 0.2 m/s (Fig. S3). In addition, a CFD simulation of a 0.01 m^2 region of this community was undertaken with the inlet and outlet assigned periodic boundary conditions (to approximate flow within an infinitely long domain) with a pressure difference of 0.2 Pa, which gave an inlet velocity of approximately 0.2 m/s.

The results demonstrated that the regions 'Subsample 2' and 'Subsample 3' were characterized by velocity profiles that differed substantially from those obtained for the full domain and 'Subsample 1' (which were influenced by the leading edge), with minimal perturbations in streamwise and vertical velocity within the community, similar to the simulation with periodic flow conditions (Fig. S3) and consistent with experimental measurements of velocity in the fully-developed region in model canopies[1–3]. 'Subsample 2' was selected for subsequent comparisons as this captured a wider area of the domain, allowing us to explore how flow velocity varied spatially within the community.

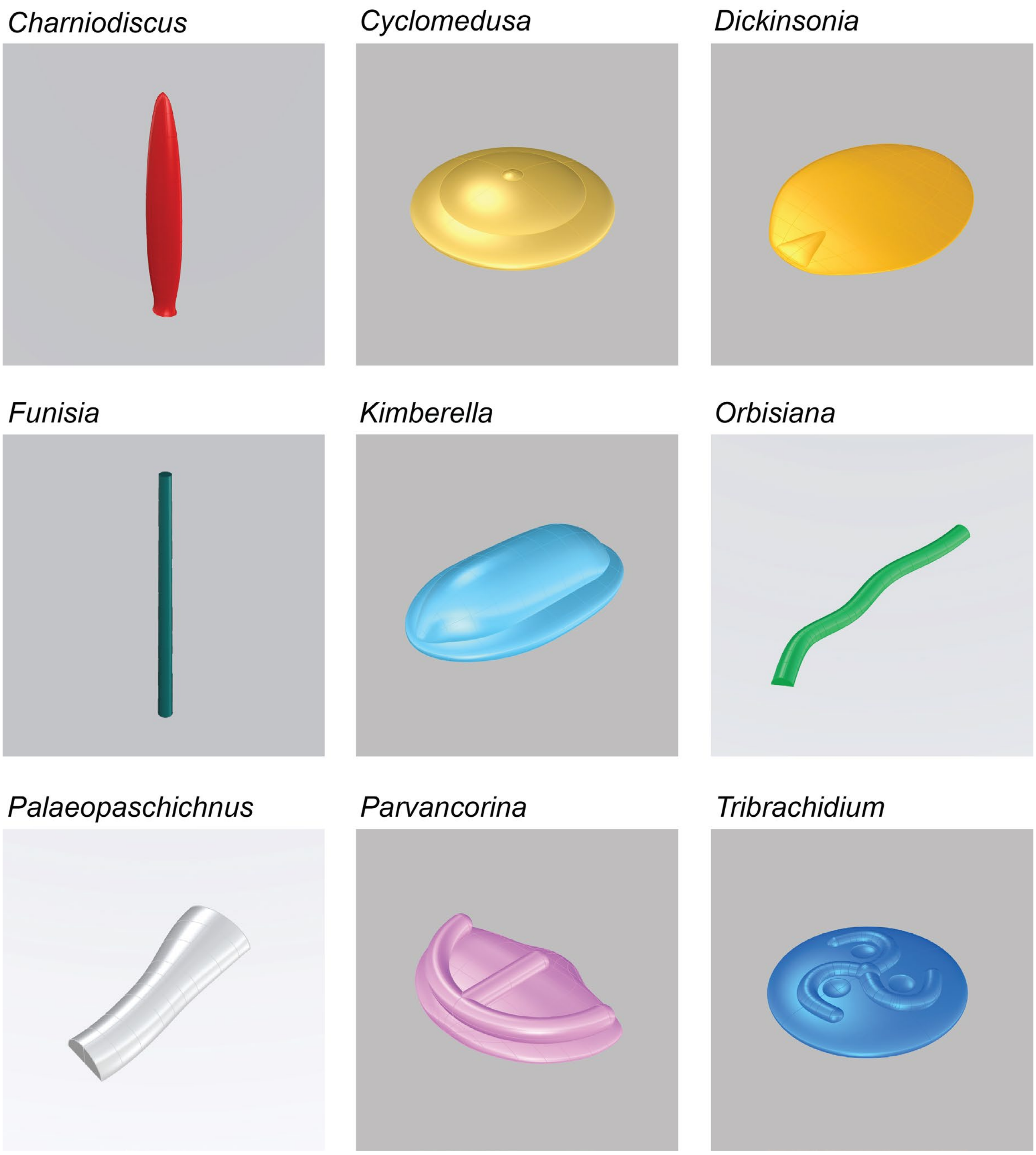

Fig. S1. Digital models used in CFD simulations. Images not to scale.

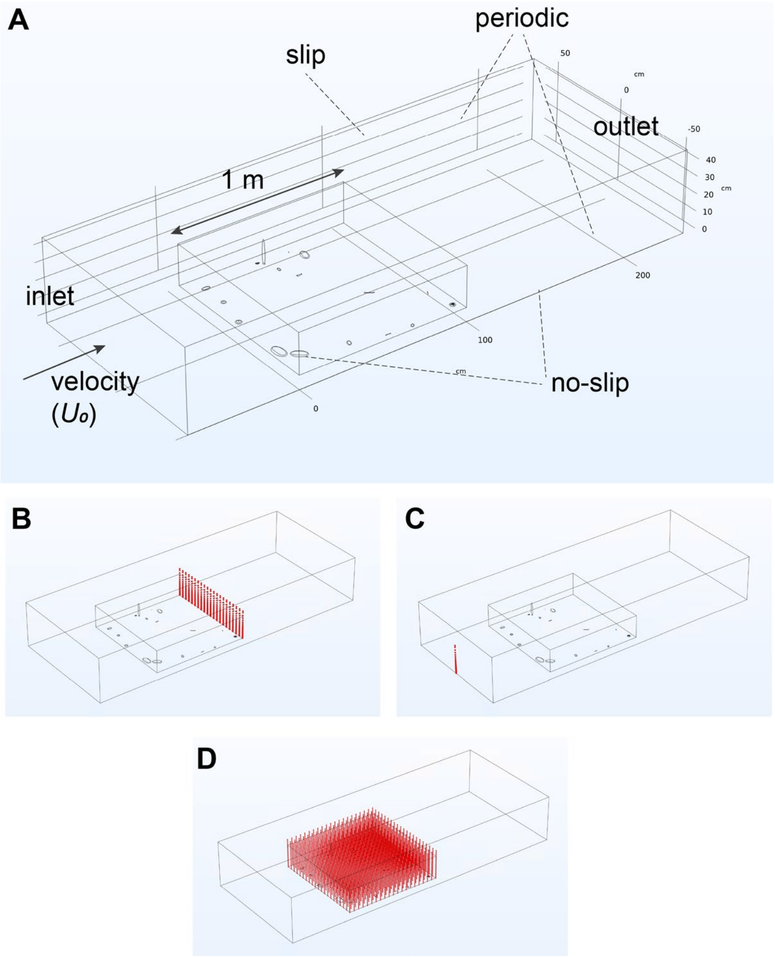

Fig. S2. Computational domain used in CFD simulations. A) Domain with boundary conditions and the direction of inlet velocity (U_0) labelled. B) Red lines showing where streamwise velocities were sampled at the back of the community. C) Red line showing where streamwise velocities were sampled at the inlet. D) Red grid of points showing where vertical velocities were sampled around the community.

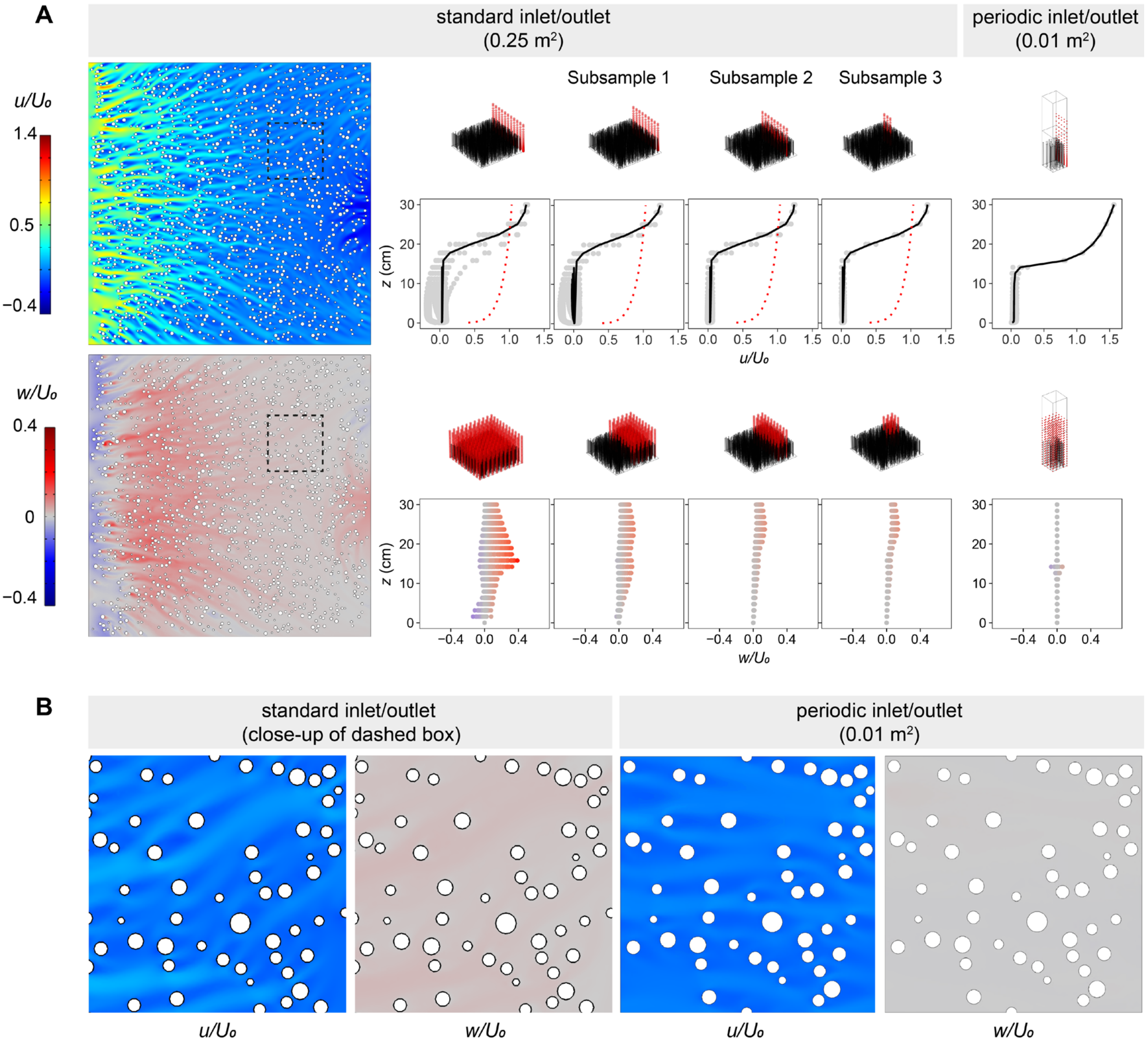

Fig. S3. Plots of streamwise and vertical velocity for different regions of one simulated community (Sim 1) of the FUN5 surface. A) Two-dimensional plots of streamwise velocity (*u*) and vertical velocity (*w*) relative to the average inlet velocity (U_0 = 0.2 m/s) for a horizontal cross-section at height *z* = 5 cm (direction of ambient flow from left to right) for the full simulated community (0.25 m^2). Plots of streamwise velocity (*u*) and vertical velocity (*w*) relative to the average inlet velocity (U_0 = 0.2 m/s) at heights between *z* = 0 to *z* = 30 cm for different regions of the full simulated community compared to a simulation undertaken for a smaller area (0.01m^2) of the community with the inlet and outlet assigned periodic boundary conditions. In plots of streamwise velocity, the black line shows the mean streamwise velocity and the dotted red line shows the undisturbed boundary layer profile. B) Two-dimensional plots of streamwise velocity (*u*) and vertical velocity (*w*) relative to the average inlet velocity (U_0 = 0.2 m/s) for the region of the full simulated community indicated by the dashed boxes in A compared to a simulation undertaken for a 0.01m^2 area with the inlet and outlet assigned periodic boundary conditions.

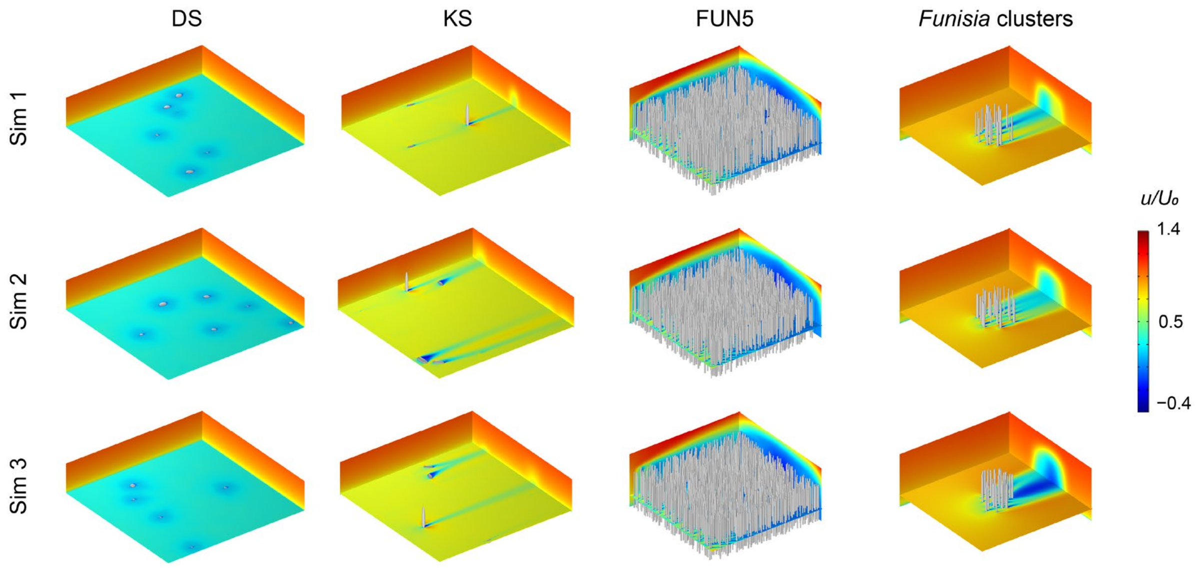

Fig. S4. Plots of streamwise velocity for White Sea communities. CFD results for three simulated communities (Sim 1–Sim 3) of the DS (1 m^2 area), KS (1 m^2 area) and FUN5 (0.25 m^2 area) surfaces and *Funisia* clusters (0.25 m^2 area). Two-dimensional plots of streamwise velocity (u) relative to the average inlet velocity (U_0 = 0.2 m/s) for horizontal cross-sections at heights z = 0.05 cm (DS), z = 1 cm (KS) and z = 5 cm (FUN5 and *Funisia* clusters) and two vertical cross-sections. Direction of ambient flow from left to right.

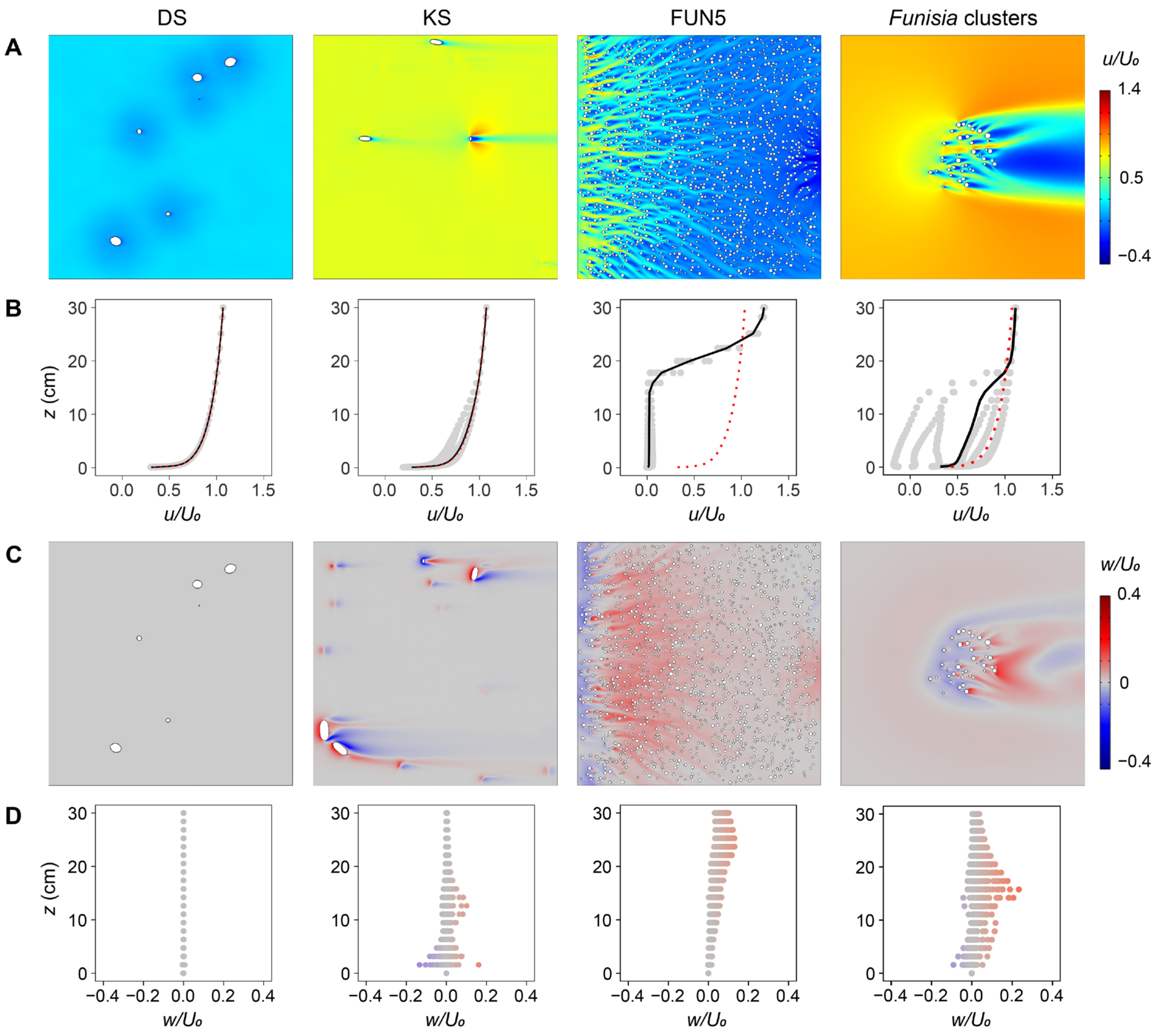

Fig. S5. Plots of streamwise and vertical velocity for White Sea communities. CFD results for one simulated community of the DS (Sim 1; 1 m^2 area), KS (Sim 1; 1 m^2 area) and FUN5 (Sim 2; 0.25 m^2 area) surfaces and *Funisia* clusters (Sim 3; 0.25 m^2 area). A) Two-dimensional plots of streamwise velocity (u) relative to the average inlet velocity (U_0 = 0.1 m/s) for horizontal cross-sections at heights z = 0.05 cm (DS), z = 1 cm (KS) and z = 5 cm (FUN5 and *Funisia* clusters). Direction of ambient flow from left to right. B) Plots of streamwise velocity (u) relative to the average inlet velocity (U_0 = 0.1 m/s) at heights between z = 0 to z = 30 cm. The black line shows the mean streamwise velocity and the dotted red line shows the undisturbed boundary layer profile. C) Two-dimensional plots of vertical velocity (w) relative to the average inlet velocity (U_0 = 0.1 m/s) for horizontal cross-sections at heights z = 0.05 cm (DS), z = 1 cm (KS) and z = 5 cm (FUN5 and *Funisia* clusters). Direction of ambient flow from left to right. D) Plots of vertical velocity (w) relative to the average inlet velocity (U_0 = 0.1 m/s) at heights between z = 0 to z = 30 cm.

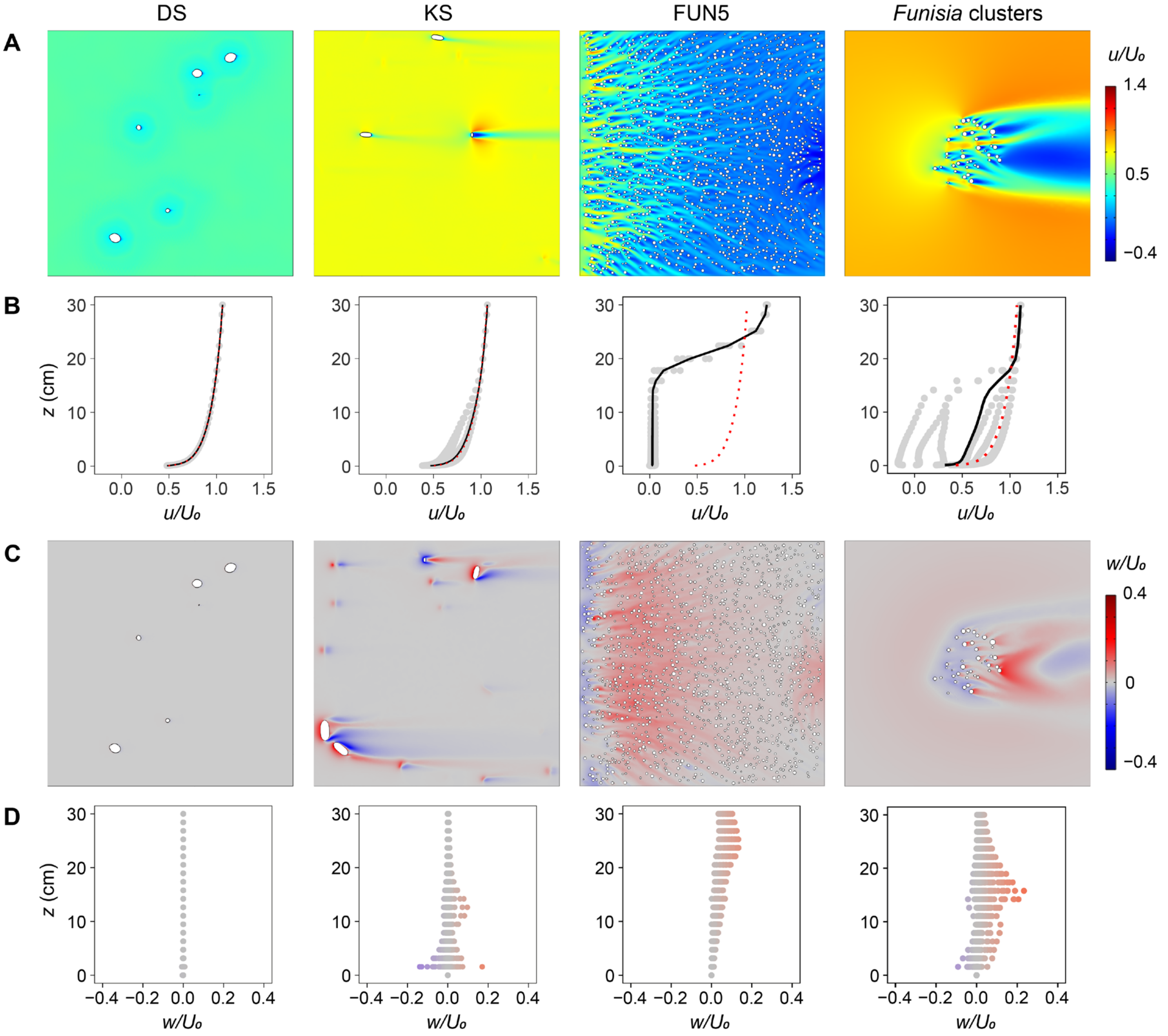

Fig. S6. Plots of streamwise and vertical velocity for White Sea communities. CFD results for one simulated community of the DS (Sim 1; 1 m^2 area), KS (Sim 1; 1 m^2 area) and FUN5 (Sim 2; 0.25 m^2 area) surfaces and *Funisia* clusters (Sim 3; 0.25 m^2 area). A) Two-dimensional plots of streamwise velocity (u) relative to the average inlet velocity (U_0 = 0.4 m/s) for horizontal cross-sections at heights z = 0.05 cm (DS), z = 1 cm (KS) and z = 5 cm (FUN5 and *Funisia* clusters). Direction of ambient flow from left to right. B) Plots of streamwise velocity (u) relative to the average inlet velocity (U_0 = 0.4 m/s) at heights between z = 0 to z = 30 cm. The black line shows the mean streamwise velocity and the dotted red line shows the undisturbed boundary layer profile. C) Two-dimensional plots of vertical velocity (w) relative to the average inlet velocity (U_0 = 0.4 m/s) for horizontal cross-sections at heights z = 0.05 cm (DS), z = 1 cm (KS) and z = 5 cm (FUN5 and *Funisia* clusters). Direction of ambient flow from left to right. D) Plots of vertical velocity (w) relative to the average inlet velocity (U_0 = 0.4 m/s) at heights between z = 0 to z = 30 cm.

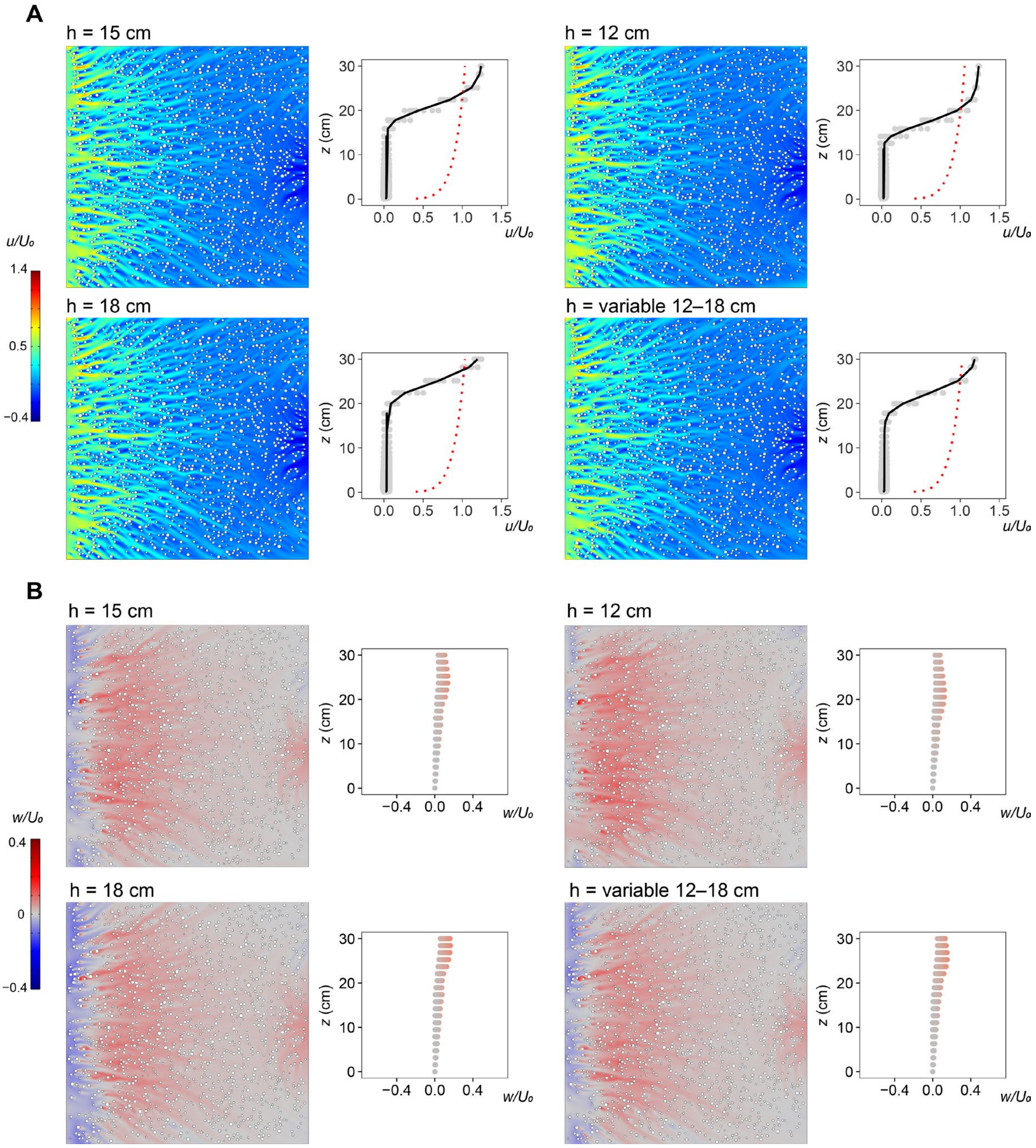

Fig. S7. Sensitivity tests of model height (h) for one simulated community (Sim 2) of the FUN5 surface (0.25 m^2 area). A) Two-dimensional plots of streamwise velocity (*u*) relative to the average inlet velocity (U_0 = 0.2 m/s) for a horizontal cross-section at height *z* = 5 cm (direction of ambient flow from left to right) and plots of streamwise velocity (*u*) relative to the average inlet velocity (U_0 = 0.2 m/s) at heights between *z* = 0 to *z* = 30 cm (the black line shows the mean streamwise velocity and the dotted red line shows the undisturbed boundary layer profile). B) Two-dimensional plots of vertical velocity (*w*) relative to the average inlet velocity (U_0 = 0.2 m/s) for a horizontal cross-section at height *z* = 5 cm (direction of ambient flow from left to right) and plots of vertical velocity (*w*) relative to the average inlet velocity (U_0 = 0.2 m/s) at heights between *z* = 0 to *z* = 30 cm.

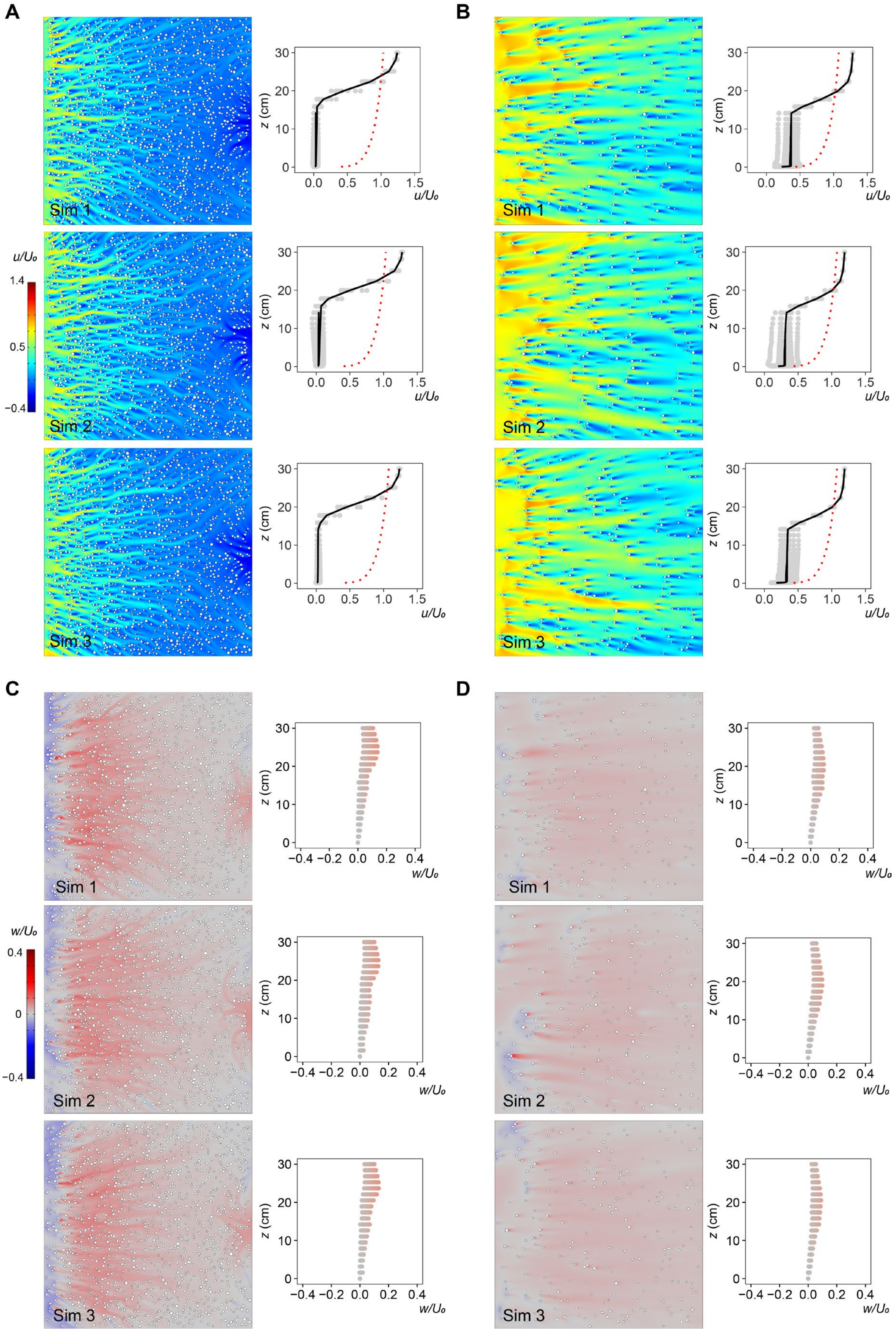

A
B
C
D
Sim 1
Sim 2
Sim 3
u/U0
1.4
0.5
−0.4
w/U0
0.4
0
−0.4
z (cm)
0
10
20
30
0.0
0.5
1.0
1.5
−0.4 −0.2 0.0 0.2 0.4

Fig. S8. Sensitivity tests of model density for three simulated communities (Sim 1–Sim 3) of the FUN5 surface (0.25 m^2 area). A, C) Original simulated communities. B, D) Low-density communities. A, B) Two-dimensional plots of streamwise velocity (*u*) relative to the average inlet velocity (U_0 = 0.2 m/s) for horizontal cross-sections at height *z* = 5 cm (direction of ambient flow from left to right) and plots of streamwise velocity (*u*) relative to the average inlet velocity (U_0 = 0.2 m/s) at heights between *z* = 0 to *z* = 30 cm (the black line shows the mean streamwise velocity and the dotted red line shows the undisturbed boundary layer profile). C, D) Two-dimensional plots of vertical velocity (*w*) relative to the average inlet velocity (U_0 = 0.2 m/s) for a horizontal cross-section at height *z* = 5 cm (direction of ambient flow from left to right) and plots of vertical velocity (*w*) relative to the average inlet velocity (U_0 = 0.2 m/s) at heights between *z* = 0 to *z* = 30 cm.

SI References

1. Moltchanov S, Bohbot-Raviv Y, Duman T, Shavit U. Canopy edge flow: a momentum balance analysis. *Water Resourc*. *Res*. 51, 2081–2095 (2015).
2. Follett E, Nepf H. Particle retention in a submerged meadow and Its variation near the leading edge. *Estuar*. *Coasts* 41, 724–733 (2018).
3. Lei J, Nepf H. Evolution of flow velocity from the leading edge of 2-D and 3-D submerged canopies. *J*. *Fluid Mech*. 916, A36 (2021).
